# The disruption of elective procedures due to COVID-19 in Brazil in 2020

**DOI:** 10.1038/s41598-022-13746-5

**Published:** 2022-06-29

**Authors:** Gustavo Saraiva Frio, Letícia Xander Russo, Cleandro Pires de Albuquerque, Licia Maria Henrique da Mota, Adriana Ferreira Barros-Areal, Andréa Pedrosa Ribeiro Alves Oliveira, João Firmino-Machado, Everton Nunes da Silva

**Affiliations:** 1 Graduate Program in Collective Health, Faculty of Health Science, University of Brasilia, Brasília, Brazil; 2 Catholic University of Pelotas, Pelotas, Brazil; 3 Department of Economics, Federal University of Grande Dourados, Dourados, Brazil; 4 Graduate Program in Medical Science, Faculty of Medicine, University Hospital of Brasília, University of Brasilia, Brasília, Brazil; 5 Graduate Program in Medical Science, Faculty of Medicine, University of Brasilia, Brasília, Brazil; 6 Graduate Program in Medical Science, Health Department of the Federal District (SES/DF), Faculty of Medicine, University of Brasilia, Brasília, Brazil; 7 Faculty of Medicine, University Hospital of Brasília, University of Brasília, Brasília, Brazil; 8 Faculty of Medicine, Institute of Public Health (ISPUP), University of Porto, Porto, Portugal; 9 Graduate Program in Collective Health, Faculty of Health Science, Faculty of Ceilândia, University of Brasilia, Brasília, Brazil

**Keywords:** Health care, Diseases

## Abstract

Elective procedures were temporarily suspended several times over the course of the pandemic of COVID-19. Monthly data from the Unified Health System (SUS) were used for the period between January 2008 and December 2020 and the interrupted time series method was used to estimate the effect of the pandemic on the number of elective surgeries and elective procedures that were not performed. Considering a 9-month period, a reduction of 46% in the number of elective procedures carried out in the SUS could be attributed to COVID-19, corresponding to about 828,429 elective procedures cancelled, ranging from 549,921 to 1,106,936. To a full recovery of pre-pandemic performance, SUS would need to increase about 21,362 hospital beds, ranging from 12,370 to 36,392 hospital beds during a 6 month-period. This effort would represent an increase of 8.48% (ranging from 4.91 to 14.45%) in relation to the total number of SUS’s hospital beds in 2019. As a result, the pandemic will leave a large number of elective procedures to be carried out, which will require efforts by health agencies to meet this demand.

## Introduction

The COVID-19 pandemic has led to an unprecedented demand for health services worldwide, challenging the capacity of healthcare systems to provide timely and high-quality care. To ensure sufficient resources to tackle the pandemic, decision makers had to reallocate hospital beds, healthcare workers (HCWs) and supplies (e.g., personal protective equipment—PPE, ventilators, and oxygen) to the COVID-19^1^ effort. Elective procedures were affected the most since they were temporally cancelled or postponed. Although it mitigated the risk of SARS-CoV-2 transmission among patients, a long-delay in recovering elective procedures may lead to physical and emotional concerns, such as increasing disease severity, reduction of quality of life, and increasing the number of avoidable deaths^2^.

Based on projections from 190 countries, estimates suggest that 72.3% of elective surgeries would be cancelled or postponed during a 12-week period of peak disruption due to COVID-19, totalling 28.4 million procedures related to colorectal, head and neck, gynaecological, plastics, upper gastrointestinal and urological surgeries performed in cancer hospitals worldwide^2^. There is also evidence indicating reduction on elective vascular^3^, orthopaedic and trauma^4,5^ surgeries. As COVID-19 pandemic is far from being solved worldwide, the hospitals’ capacity to deliver elective procedures may continue to be constrained to avoid nosocomial infection, even in periods when there is a declining number of COVID-19 cases and deaths^6^. On this basis, it is important to bring robust estimates of the number of elective procedures cancelled or postponed informing recovery plans by health systems.

Brazil provides a unique opportunity to study this issue. First, the Brazilian government has recommended to temporarily suspend elective procedures several times over the course of the pandemic. Second, Brazil is an upper-middle income country, with large population (over 211 million inhabitants), with extensive and entrenched health and socioeconomic inequalities across the country^7^, which have affected states and municipalities in different ways. Third, as of June 25th, 2021, Brazil ranks third and second in number of cases (behind the USA and India) and deaths (behind the USA) for COVID-19 worldwide, respectively^8^. The second wave of COVID-19 in Brazil, which started in November 2020, was devastating, with several cities reaching more than 90% occupancy for intensive care units and shortage of drugs and oxygen^9^. Finally, 75% of the Brazilian population receive healthcare only through the public health system^10^.

Our study aims first to estimate the total number of elective procedures cancelled or postponed due to the COVID-19 pandemic during a 9-month period (from April to December 2020), and then aims to use these estimates to predict the additional supply of hospital beds needed to recover the frequency of elective procedures in the public health system in Brazil. Our analyses were based on the interrupted time series approach, with monthly data from 2008 to 2020 period.

## Methods

### Study setting

Brazil has provided universal and comprehensive care, free of charge at point of service, through the Unified Health System (SUS, Sistema Único de Saúde) since 1988^11^, allowing all citizens to have access to health and adequate treatment, including for COVID-19. In December 2020, there were 7035 hospitals covering all of SUS. These hospitals performed 8,747,734 emergency hospitalizations in 2020, down from 9,501,285 hospitalizations performed in 2019, a reduction of 7.93%. Regarding the elective procedures, the same hospitals performed 2,527,445 and 1,587,418 hospitalizations in 2019 and 2020, respectively, a reduction of 37.19%.

Since February 26, 2020, when Brazil registered the first case of COVID-19, there has been 18,169,881 confirmed cases and 507,109 deaths caused by the disease in the country, according to data reported as of June 25th, 2021, by the World Health Organization (WHO)^8^. Although Brazil has experienced a rapid spread of cases and deaths for COVID-19, it was hit in an unequal way across the country and over time^12^. To increase the number of hospital beds for COVID-19, it was recommended that elective surgeries should be suspended^13^ since the beginning of the pandemic, and this strategy has become usual in times of resurgence of the pandemic in states and municipalities.

### Study design

We used Interrupted Time Series (ITS), a quasi-experimental approach, to estimate the difference between two periods (before and after the start of the COVID-19 pandemic in Brazil) in terms of the hospitalization rate for elective procedures per 100,000 inhabitants. The unit of analysis is the hospital admission and covers data from all over Brazil through the Hospital Information System (SIH) of SUS, with monthly data from January 2008 to December 2020. The series was interrupted in March 2020, with the first death by COVID-19 in Brazil and when community transmission was announced^14^.

### Variables and sources

Our dependent variable is the rate of hospitalization for elective procedures per 100,000 inhabitants. Our main source is the SIH/SUS^15^, from which we collected the number of hospitalizations for elective procedures per month. SIH/SUS covers all inpatient care provided by hospitals to SUS, which is used for reimbursement purposes. In SIH/SUS, procedures are classified as emergency and elective, with the latter accounting for around 20% of the total number of hospitalizations in SUS. We considered two sets of elective procedures performed in SUS. The first refers to the so-called general surgeries, which include procedures such as hernioplasties, excisions, some oncological procedures, among others. General surgeries are the most common surgical procedures performed in SUS, representing around 20% of total elective procedures. The second refers to all elective procedures, including the general surgeries, ophthalmology, gynecology, urology, among others. We also used data from the Brazilian Institute of Geography and Statistics (IBGE, Instituto Brasileiro de Geografia e Estatística)^16^, from which we collected data on the number of inhabitants per month. Data from IBGE were used to calculate the rate for 100,000 inhabitants in Brazil. All the data used are freely available for download. The procedures performed in this study are in accordance with the relevant guidelines and regulations.

### Statistical analyses

The ITS model is used when there are no (or few) control groups but a series of data which can be divided into before and after the intervention. As there is no control group, the model used here is called “single group”. The empirical strategy provides four parameters that can be used to understand the variable over time. First, the “intercept” captures the initial outcome level (hospitalization rate for elective procedures in January 2008). Second, the “baseline trend” captures the pre-intervention trend (hospitalization rate trend from January 2008 to March 2020). Third, the “level change” captures the change in the level of the outcome after the intervention (hospitalization rate for elective procedures in April 2020) after the exposure. Fourth, the “trend change” captures the change in the slope after the intervention (hospitalization rate trend from April 2020 to December 2020)^17^.

We identified that our dependent variable contains seasonal variation, since the hospitalization rate for elective procedures varies over the months of the year. On this basis, we used the classical decomposition method for removing the seasonal patterns of the time series. We also investigated residual autocorrelation by using the Cumby-Huizinga test^18^. We identified autocorrelation at lag 4 for general surgeries and all elective procedures. For the regression analyses, we used ordinary least square (OLS) regression with Newey-West standard errors. We used the ITSA package for regressions and the command ACTEST for autocorrelation, both using STATA 14.2.

### Scenario analyses

We conducted scenario analyses to predict the additional supply of hospital beds needed to recover elective procedures in SUS. These analyses consist of providing a set of plausible future events that may influence the response of SUS in meeting the cancelled or postponed demand of elective procedures in Brazil. Scenarios combine three perspectives: (i) optimist, corresponding to the most favourable parameters to SUS; (ii) moderate, considering the baseline parameters (normally, the average measure); and (iii) pessimist, representing the least favourable parameters to SUS. We also considered three periods by which SUS would fully meet the cancelled or postponed demand for elective procedures (6, 12 and 24 months). Additional hospital beds were calculated for: (i) elective general surgeries; and (ii) all elective procedures, including general surgeries.

The number of elective procedures cancelled or postponed in 2020 was calculated using the ITS results. Coefficients were used to calculate the number of elective procedures performed monthly in the period from 2008 to 2020. The intercept (initial level) and the baseline trend were used to forecast the number of elective procedures that would be performed in a scenario under normal conditions (without COVID-19 pandemic) for the period from April to December 2020. The hospitalization rate for elective procedures cancelled or postponed is the difference between the predicted number of elective procedures (with pandemic) and the number forecasted in the scenario under normal condition (without pandemic). We used the lower and upper limits calculated from the confidence interval (95% Confidence Interval—CI) to represent optimist and pessimist scenarios, respectively. As average length of stay varies among elective procedures, we used 2019 hospital admissions from SIH/SUS to estimate the average (moderate), lower (optimist) and upper (pessimist) limits for general surgeries and all elective procedures. Another key issue is the hospital bed occupancy rate, which measures the utilization of the available bed capacity (percentage of beds effectively occupied by patients in a year). We used data from the literature to define bed occupancy rate for optimist (95%), moderate (85%) and pessimist (75%)^19^. We also calculated the additional supply of hospital beds as a share of total number of hospital beds in SUS, excluding hospital-day and paediatric beds, totalling 251,874 beds in December 2019.

## Results

SUS performed 27,735,937 elective procedures from 2008 to 2020. Surgeries are the most frequent elective procedures in SUS (77.68%), followed by clinical treatments (20.78%), transplants (0.93%), and diagnoses (0.61%). For elective surgeries, we also considered a subset of operations that presented a higher frequency of cases (called general surgeries), corresponding to 19.78% of all elective procedures performed by SUS in the same period (Table 1).

**Table 1:** Frequency of the elective procedures by type of procedure, Brazil, 2008–2020.

Type of procedure	Number of procedures (2008–2020)	%
**Surgical**	21,545,588	77.68
General surgery		
Cholecystectomy; hernioplasty; excision and suture/graft; gastric bypass; debridement of ulcer/devitalized tissue; dermoid cyst excision; multiple skin lesion or subcutaneous cell tissue removal in oncology; resection of soft tissue tumor in oncology; multiple intra-abdominal biopsies in oncology; laparoscopic inguinal herniorrhaphy; repair of other hernias; laparoscopic umbilical herniorrhaphy; removal and suppression of skin lesion and subcutaneous cell tissue; implantation of semi- or totally implantable indwelling catheter main procedure; exploratory laparotomy)	5,489,021	19.79
Other		
Treatment with multiple surgeries; Hysterectomy; Varicose vein surgery; Circumcision; Tubal ligation; Tonsillectomy; Phacoemulsification; Sequential procedures in oncology; Vasectomy; Colpoperineoplasty; Hemorrhoidectomy; Removal of intraosseous wire or pin; Angioplasty; Sectorectomy/quadrantectomy; Oophorectomy/oophoroplasty; Surgical treatment of compression syndrome in osteofibrous tunnel at the level of the carpus; others	16,056,567	57.89
**Clinicians**	5,762,309	20.78
Treatment in psychiatry; treatment for neurological disorders; cancer patient treatment; others		
**Diagnostic purpose**	168,906	0.61
Biopsy; polysomnography; video-electroencephalogram; tracheoscopy; video-thoracoscopy; others		
**Organ, tissue and cell transplants**	259,134	0.93
Total	27,735,937	100.00

Table 2 presents the results from the empirical strategy by means of ITS. The hospitalization rate for elective general surgeries has its initial level at 15.184 per 100,000 inhabitants (95% CI 14.725 to 15.643; *p* < 0.001), with a positive trend of 0.038 per 100,000 inhabitants per month (95% CI 0.030 to 0.045; *p* < 0.001). In March 2020, however, the COVID-19 pandemic breaks out, so in April there is a drop of 16.687 procedures per 100,000 inhabitants (95% CI − 19.045 to − 14.328; *p* < 0.001) and a new upward trend of 1.002 procedures per 100,000 inhabitants (95% CI 0.615 to 1.390; *p* < 0.001). The hospitalization rate for all elective procedures follows the same pattern as the general surgeries, with all coefficients significant at the 1% level (Table 2).

**Table 2:** Results from the interrupted times series approach for hospitalization rate for elective procedures (general surgeries and all elective procedures) in Brazil, January 2008 to December 2020.

Variable	General surgeries		All elective procedures	
	Coefficient	*p* value	Coefficient	*p* value
Intercept	15.184 (14.725 to 15.643)	< 0.001	85.636 (83.554 to 87.720)	< 0.001
Baseline Trend	0.038 (0.030 to 0.045)	< 0.001	0.062 (0.028 to 0.096)	< 0.001
Level change after Covid-19	− 16.687 (− 19.045 to − 14.328)	< 0.001	− 57.850 (− 66.430 to − 49.270)	< 0.001
Trend change after Covid-19	1.002 (0.615 to 1.390)	< 0.001	3.630 (2.122 to 5.138)	< 0.001

Confidence intervals in Parentheses. All elective procedures include general surgeries.

**Figure 1:**
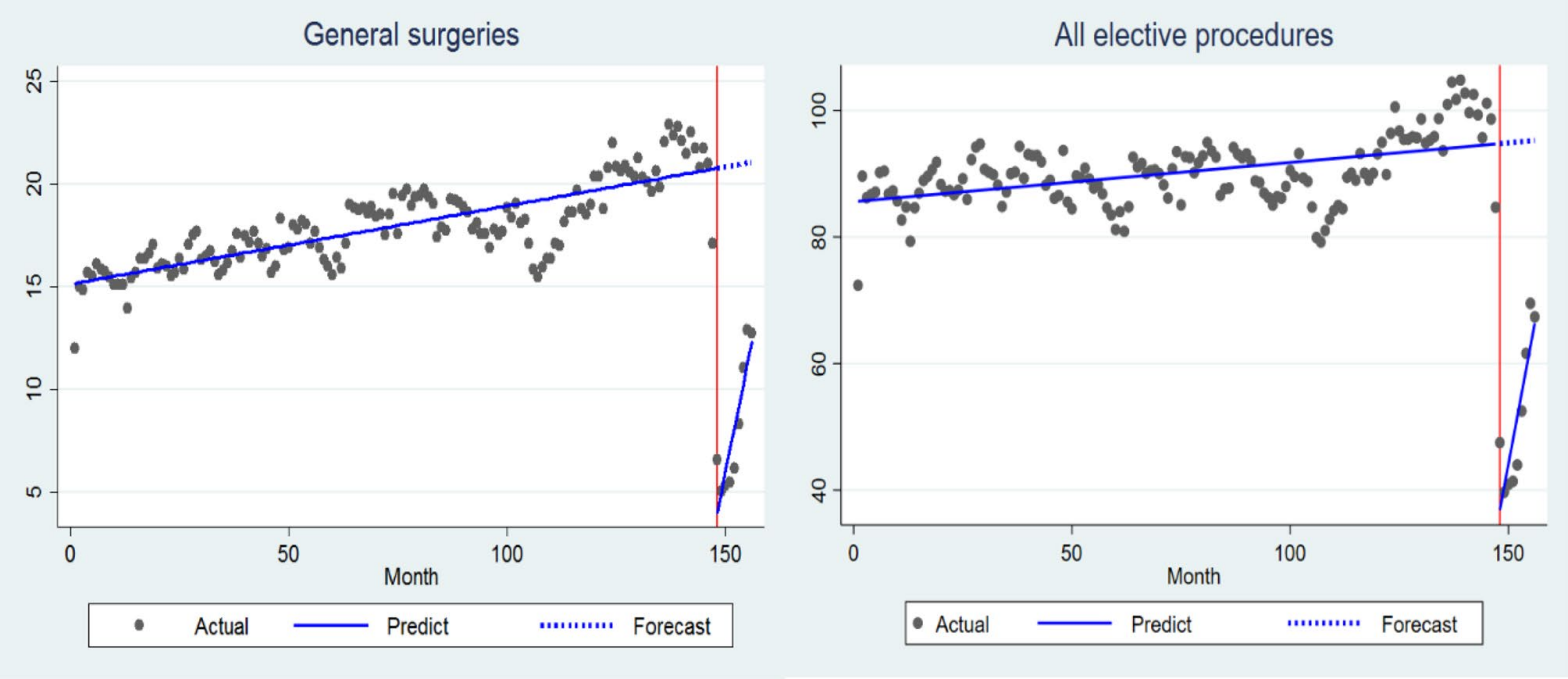
Graphical representation of the effect of COVID-19 pandemic on hospital rate for elective procedures (general surgeries and all elective procedures) from interrupted time series regression in Brazil, 2008–2020.

Figure 1 graphically presents the trend of elective procedures over time. It is worth noting that the hospitalization rate for general surgeries per 100,000 inhabitants was 21.0 in February 2020, reaching the lowest value in May (5.1 per 100,000 inhabitants). During the pandemic, the hospitalization rate for all elective procedures dropped from 98.6 to 39.7 per 100,000 inhabitants in February and May 2020, respectively.

Based on ITS results, there would be 828,429 elective procedures cancelled or postponed due to COVID-19 pandemic in the period of Apr-Dec/2020, ranging from 549,921 (optimist scenario) to 1,106,936 (pessimist scenario). Of them, around 30% were general surgeries. The transition from a temporary suspension of elective procedures to a full recover of pre-pandemic performance, SUS would need to increase 21,362 hospital beds, ranging from 12,370 (optimist) to 36,392 (pessimist) hospital beds during a 6 month-period. This effort would represent an increase by 8.48% (ranging from 4.91 to 14.45%) in relation to total number of SUS hospital beds in 2019. Considering a large period to reach a full recover, the effort would represent an increase of 2.12% (ranging from 1.23 to 3.61%) during a 24 month-period (Table 3).

**Table 3:** Scenarios for the supply of additional hospital beds in the public health system (SUS) to overcome the cancelled or postponed general surgeries and all elective procedures in 2020, Brazil.

Parameters	General surgeries			All elective procedures		
	Optimistic	Moderate	Pessimist	Optimistic	Moderate	Pessimist
**To overcome in 6 months**						
Number of hospital admission cancelled or postponed	168,759	242,770	316,783	549,921	828,429	1,106,936
Average length of stay (days)	1.5	1.6	1.7	3.9	4.1	4.5
Hospital bed occupancy (%)	95	85	75	95	85	75
Number of additional hospital beds	1460	2504	3934	12,370	21,362	36,392
Percentage in terms of total number of SUS’s hospital beds	0.58	0.99	1.56	4.91	8.48	14.45
**To overcome in 12 months**						
Number of hospital admission cancelled or postponed	168,759	242,770	316,783	549,921	828,429	1,106,936
Average length of stay (days)	1.5	1.6	1.7	3.9	4.1	4.5
Hospital bed occupancy (%)	95	85	75	95	85	75
Number of additional hospital beds	730	1252	1967	6185	10,681	18,196
Percentage in terms of total number of SUS’s hospital beds	0.29	0.50	0.78	2.46	4.24	7.22
**To overcome in 24 months**						
Number of hospital admission cancelled or postponed	168,759	242,770	316,783	549,921	828,429	1,106,936
Average length of stay (days)	1.5	1.6	1.7	3.9	4.1	4.5
Hospital bed occupancy (%)	95	85	75	95	85	75
Number of additional hospital beds	365	626	984	3093	5340	9098
Percentage in terms of total number of SUS’s hospital beds	0.14	0.25	0.39	1.23	2.12	3.61

The number of hospital admissions unattended in 2020 was based on uninterrupted time series’ results, with optimistic, moderate and pessimist scenario based on lower-limit of the 95% confidence interval, average effect, and upper-limit of the 95% confidence interval, respectively. The average length of stay was based on monthly data from 2019, using the same rationale for defining scenario as described in the case of number of hospital admission unattended. The number of months to overcome the unattended procedures was defined arbitrarily. The hospital bed occupancy measures the percentage of beds that in fact was used during the period analysed; occupancy lower than 100% can be explained by hospital management failure, patient desistance or clinically unwell; the Brazilian Ministry of Health recommend an occupancy rate from 75 to 85%, which we used as pessimist and moderate scenario, respectively^19^. We used the total number of hospital beds in SUS on December 2019, totalling 294,968 hospital beds in Brazil.

## Discussion

Our study showed that COVID-19 had a devastating effect on elective procedures in the Brazilian public healthcare system (SUS), with an abrupt reduction of these procedures in the very beginning of the pandemic. Considering a 9-month period (Apr-Dec/2020), a reduction of 46% was attributed to COVID-19, corresponding to 828,429 elective procedures cancelled or postponed, ranging from 549,921 to 1,106,936. It is worth noting that this proportion reached a reduction of 57% in the very beginning-period of the pandemic (Apr–Jun/2020). We also predicted the number of additional hospital beds required to fully attain the number of elective procedures cancelled or postponed due to COVID-19 in 2020. To overcome it in the short-term (6 months), the SUS would need to increase its hospital bed capacity by 8.48% (21,362 additional hospital beds), ranging from 4.91% (12,370) to 14.45% (36,392). A lower incremental capacity would be required if the period to fully recovery elective procedures from 2020 increases. However, this would happen in detriment of patient’s health, since elective means nonemergency procedure, but it is still necessary and cannot be postpone for a long-term.

A study provided estimates of the global burden of elective surgeries for 190 countries, based on expert surgeons’ perception and modelling approach. Brazil would account to around 10% of surgery projected to be cancelled during a peak of 12-weeks of disruption due to COVID-19 (2.97 million in Brazil of 28.4 million in 190 countries)^2^. This estimate seems to overestimate the elective burden in Brazil, even considering our upper limit confidence interval, which predicted 1.1 million elective procedures cancelled due to COVID-19 for a period of 39 weeks in SUS, including the peak of cases and deaths of COVID-19 in the first wave of the disease in the country. Our estimates were based on real-world data and included all elective procedures defined by the Ministry of Health.

Most of the studies conducted in Brazil are descriptive analysis, not accounting for causal effects between the reduction of elective procedures and the COVID-19 pandemic. That said, a descriptive study compared the average number of elective procedures from 2016 to 2019 with 2020, suggesting a reduction of 34.82% (95% CI 34.73–34.90)^20^. Another descriptive study conducted in the state of Ceará, Brazil, found a reduction of 89.3 and 67.9% in the number of transplants and organ donations, respectively, comparing Apr-Jun/2020 with Apr-Jun/2019^21^. An analytical study used Autoregressive Integrated Moving Average (ARIMA) models to estimate the surgical backlog in Brazil, based on the Brazilian public health system monthly records of surgical procedures from Jan/2016 to Dec/2020. Estimates suggested that 1,119,433 (CI 95% 762,663–1,523,995) elective surgeries were backlogs from March to December 2020^22^.

International evidence has also highlighted the negative consequences of the COVID-19 pandemic on elective procedures. In England, the number of elective surgeries has not yet reached the pre-pandemic volume in the National Health Service, and it may worsen with the Omicron variant^23^. A study conducted in the Netherlands calculated the net monetary losses per week of delayed elective surgical procedures. Considering that 30% of 13 elective procedures were delayed for 3 months, it would result in €0.3 million extra costs for the healthcare system and a total impact on both cost and quality of life of €3.6 million (net monetary loss)^24^.

Hospital bed capacity is not only limited by the number of beds per se, but also by monitoring devices, life supporting machines and high-quality trained HCWs^25^. In Brazil, there is an entrenched asymmetrical distribution of HCWs across the country, skewed towards municipalities with higher technological and socioeconomic status. South and Southeast regions have higher density of HCWs (surgeons, anaesthesiologists, and obstetricians) than North and Northeast (the poorest regions in Brazil)^26^. Another key-disparity is the distribution of HCWs between public and private systems. A study indicated that there is a rising trend for physicians and nurses in the private health insurance sector than in the public health system in Brazil from 2005 to 2016; moreover, the states with the lowest increasing trends for physicians are in the less developed regions of the country (North and Northeast), mostly due to lack of infrastructure in health facilities and few professional training institutions^27^. Other studies have also highlighted the shortage of speciality services as one of the main concerns in SUS, leading to unmet demand, long waiting lists, delay in diagnosis and poor prognosis^10,28^. Considering all the above issues, the recovery plan for elective procedures in Brazil can face numerous challenges.

There is a vast literature on the unintended consequences of delaying elective surgeries, particularly by reducing quality of life and overall health status, worsening disease prognosis, and increasing mortality^29,30^. For joint replacement surgery, a moderate delay could result in muscle wasting due to immobility and exacerbate comorbities, posing more challenges for rehabilitation. Moreover, patients with unremitting pain tend to be more likely to use opioids, alcohol, and illegal drugs^31^. A literature review searched for studies that assessed the effects of delayed general elective surgery in terms of physical, psychological, and social aspects^32^. Long waiting times for varicose vein surgery and inguinal hernia repair involve marginal physical, psychological or social suffering, and severe deterioration is unlikely. The impact of delaying cholecystectomy seems to be more profound by exacerbating negative effects on the three dimensions analysed (physical, psychological or social aspects). Risk of emergency admissions or complications was higher for patients with longer waiting times for cholecystectomy than for short waits. The authors concluded that there is a remarkable paucity of studies addressing the effect of waiting time for elective surgery on patients for all disorders analysed^32^.

Elective recovery plans will demand a large effort from public and private sectors. Several strategies have been implemented worldwide. Many countries have adopted lockdowns and restrictions in places where people gather such as shops, restaurants, as well as closure of educational institutions, flattening the curve of cases by curbing the spread of the virus^33^. In China, new hospitals were built in a very short-term, increasing the number of hospital beds available to population^25^. In Brazil, the SUS has increased around 18,000 hospital beds for COVID-19 during 2020. However, most of them were beds from field hospitals (*hospitais de campanha*), which acts as a temporary health facility for COVID-19. For example, there were 13,011 field hospital beds in June 2020, which dropped to 9334 beds in December 2020, a reduction of 29%^15^. Then, it is likely that these new beds from field hospitals are closed after a sharp drop in cases and hospitalizations for COVID-19. On this basis, it shrinks the hospital capacity for elective procedures recovery plan in Brazil.

### Strengths and weaknesses of this study

We have addressed several methodological issues related to previous studies. First, we used a rich administrative database, by which real-world data is recorded by means of reimbursement. Second, we accounted for causal effect, by using a quasi-experimental approach through ITS. Third, we provided estimates on the additional hospital beds needed to a fully recovery plan for elective procedure in Brazil, based on the best available parameters.

That said, it is also worth noting our limitations. First, we considered real-world data for elective procedures (i.e., what in fact were performed by SUS pre- and post-exposure to COVID-19 pandemic). On this basis, we did not include patients from the waiting list to receive any elective procedure, which could suggest we are underestimating our results. Second, there is a delay between 3 and 6 months for inpatient records being released in the hospital information system (SIH/SUS). Thus, our data just marginally reflects the start of the second wave of COVID-19 in Brazil, which affected locally the municipality of Manaus, in the Amazon region, in November 2020. The second wave led to the saturation of the hospital capacity in several large municipalities across the country at the beginning of 2021. Thus, the problem of temporarily suspending the elective procedure may worsen in 2021, at least at the beginning of the year (Jan-Mar/2021). Third, we have not controlled for confounders, such as socioeconomic status, prevalence of diseases, and number healthcare workers since these data are not available per month and over the period analysed (2008–2020).

### Public health implications

We provided real-world estimates for the disruption of elective procedures due to COVID-19 pandemic in Brazil. During a 9-month period in 2020, SUS has cancelled or postponed 828,429 elective procedures, which could reach 1,106,936 in the pessimist scenario for the same period. We also discussed the devastating long-term consequences of delaying elective surgeries in terms of clinical and emotional aspects. This combination of factors lead us to claim this public problem as an epidemic of chronic diseases within the pandemic of COVID-19.

Additional hospital capacity will be required to effectively implement elective recovery plans by SUS across the country. In 2020, the main strategy to increase hospital beds conducted by Brazilian governments was by means of field hospitals, which will close as COVID-19 outbreak slows down. This scenario tends to become worse due to austerity policies that have been adopted since 2015, culminating with the Constitutional Amendment 95 (EC95) in 2016, by which no real growth in federal expenditures is allowed for the next 20 years. The federal government raised more money to tackle COVID-19 pandemic through domestic debt, which was approved by the Nacional Congress. We are afraid that elective recovery plan will not have the same political appeal as COVID-19 did. Based on that, it is important to highlight this problem to decision makers, researchers, and media, since the epidemic of chronic diseases could become the worst and last wave of the COVID-19 pandemic.

Last but not least, the elective recovery plan will need a systematic and continuous cooperation among all levels of governments in Brazil (federal, state and municipal), by strengthening the health care networks. About 40% of the Brazilian municipalities do not have any hospital beds, which will require access to hospitals from other municipalities within a health region or state.

## Conclusions

Our findings suggested that COVID-19 was associated with a reduction of 46% of elective procedures during the first 9 months (Apr–Dec/2020) of the pandemic in Brazil, representing 828,429 (CI 95% 549,921–1,106,936) elective procedures cancelled or postponed. To overcome this problem in the short-term (6 months), the public health system would need to increase by 8.48% the total number of hospital beds, corresponding to 21,362 additional hospital beds, ranging from 4.91% (12,370) to 14.45% (36,392).
